# Phylogenetic Characterization of Marine Benthic Archaea in Organic-Poor Sediments of the Eastern Equatorial Pacific Ocean (ODP Site 1225)

**DOI:** 10.3390/microorganisms4030032

**Published:** 2016-09-06

**Authors:** Antje Lauer, Ketil Bernt Sørensen, Andreas Teske

**Affiliations:** 1 Department of Marine Sciences, University of North Carolina at Chapel Hill, Chapel Hill, NC 27599, USA; alauer@csub.edu (A.L.); kebs@ramboil.com (K.B.S.); 2 Biology Department, California State University Bakersfield, Bakersfield, CA 93311-1022, USA; 3 Ramboll, Copenhagen DK-2300, Denmark

**Keywords:** Marine archaea, Marine Group I, subsurface, marine sediment, Ocean Drilling Program

## Abstract

Sequencing surveys of microbial communities in marine subsurface sediments have focused on organic-rich, continental margins; the database for organic-lean deep-sea sediments from mid-ocean regions is underdeveloped. The archaeal community in subsurface sediments of ODP Site 1225 in the eastern equatorial Pacific (3760 m water depth; 1.1 and 7.8 m sediment depth) was analyzed by PCR, cloning and sequencing, and by denaturant gradient gel electrophoresis (DGGE) of 16S rRNA genes. Three uncultured archaeal lineages with different depth distributions were found: Marine Group I (MG-I) within the Thaumarchaeota, its sister lineage Marine Benthic Group A (MBG-A), the phylum-level archaeal lineage Marine Benthic Group B (also known as Deep-Sea Archaeal Group or Lokiarchaeota), and the Deep-Sea Euryarchaeotal Group 3. The MG-I phylotypes included representatives of sediment clusters that are distinct from the pelagic members of this phylum. On the scale from fully oxidized, extremely organic carbon-depleted sediments (for example, those the South Pacific Gyre) to fully reduced, organic carbon-rich marine subsurface sediments (such as those of the Peru Margin), Ocean Drilling Program (ODP) Site 1225 falls into the non-extreme organic carbon-lean category, and harbors archaeal communities from both ends of the spectrum.

## 1. Introduction

Molecular surveys of microbial communities in marine subsurface sediments have detected distinct archaeal populations in deep-sea sediments of the central oceanic basins, and near-shore or continental margin locations; these differences most likely reflect the contrasting redox states and organic carbon content of organic-lean, oxidized open-ocean sediments with low sedimentation rates, and organic carbon-rich, reduced coastal and continental margin sediments that receive primary production from the water column and terrestrial input [1,2]. Low cell numbers and poor DNA recovery have limited sequence-based microbial community analyses of organic-lean, open-ocean sediments to relatively few case studies [3,4,5,6]. In consequence, fundamental aspects of these benthic microbial ecosystems—phylogenetic composition, functional gene repertoire, and metabolic activities—are poorly known.

**Figure 1:**
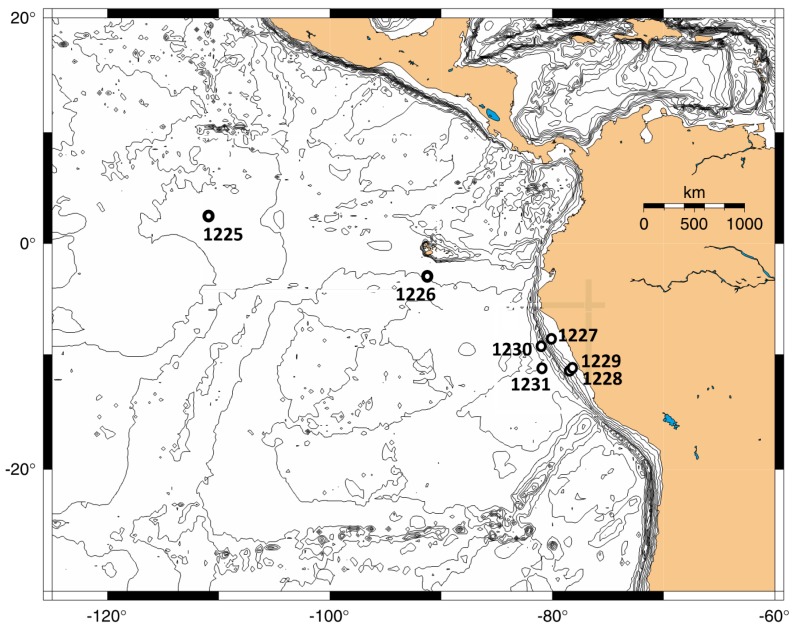
ODP Site 1225 in the eastern equatorial Pacific, and other drilling sites of ODP Leg 201 near Galapagos (1226), on the Peru Margin (1227 to 1229), in the Peru Trench (1230), and Peru Basin (1231). Modified from [7].

The first Ocean Drilling Program (ODP) expedition dedicated to the study of life deep beneath the seafloor, ODP Leg 201, combined detailed geochemical analyses, cell counts, cultivations, and molecular screening of subsurface microbial communities [7], and succeeded in a comprehensive census of subsurface microbial life in the context of geochemical controls that shape microbial community composition, abundance, and activity [8,9,10,11,12,13,14]. Sampling sites were selected that represented low-activity sediments from central oceanic basins as well as organic-rich coastal sediments from upwelling areas (Figure 1). Subsurface sediment cores were obtained from open ocean sites with organic-poor sediments in the eastern equatorial Pacific (Sites 1225 and 1226) and in the Peru Basin (Site 1231). These sites had cell densities in the range of 10^5^ to 5 × 10^6^ cells/cm^3^ below the upper few meters of the sediment column, low organic C content (1%–2% for ODP Site 1226; <0.4% for ODP Sites 1225 and 1231), near-linear or slowly changing profiles of sulfate and methane, indicating little sulfate depletion or methane accumulation, and considerable depth penetration of the highly dynamic electron acceptor nitrate [8]. Contrasting cores were obtained from organic-rich sediments on the Peru Margin (Sites 1227, 1228, 1229), and from organic-rich deep-sea sediments in the Peru Trench (Site 1230) [7]. These sites were characterized by total organic carbon (TOC) in the range of 2.5%–5%, prokaryotic cell counts mostly between 5 × 10^6^ and 10^8^ cells/cm^3^ in the subsurface sediment, and steep porewater sulfate and methane gradients indicating downcore sulfate depletion and methane buildup [8].

ODP Site 1225 offered an opportunity to explore subsurface microbial communities in organic-poor, oxidized sediments that contrast to organic-rich, reduced subsurface sediments that have been the focus of most 16S rRNA-based microbial community analyses. The archaeal community analyses from Site 1225 have been abstracted and discussed in comparative biogeographical surveys and overview chapters on subsurface microbiology [1,11,15,16,17], but have never been published in a dedicated study. Here we report the archaeal 16S rRNA gene sequencing survey of ODP Site 1225 in the eastern equatorial Pacific, document the variable outcomes of different DNA extraction methods, and provide a detailed phylogenetic analysis. We are also re-examining ODP Site 1225 in the context of more recently studied oligotrophic open-ocean sediments of the subtropical gyres in the South Pacific and the North Atlantic Oceans.

## 2. Material and Methods

**Site description.** ODP Site 1225 (Figure 1) is located in the eastern equatorial Pacific (2°36.25 N, 110°34.29 W) at 3760 m water depth near the present day boundary of the South Equatorial Current and the North Equatorial Countercurrent [18].

The clay-rich sediments of this site, consisting of carbonate and siliceous nannofossil oozes and chalk, are representative for a large portion of the central Pacific Ocean. The sediment layer at Site 1225 was 320 m thick and its age ranged from the Pleistocene (0–27 m below surface (mbsf), 1.8 Ma) to the Pliocene (28–98 mbsf, 5.2 Ma) and the Miocene (99–320 mbsf, 11.5 Ma). The water depth (3760 m) at Site 1225 reduces the input of photosynthetically derived organic material; shipboard measurements of total organic carbon (TOC) showed low values (≤0.2% to 0.42%) throughout Site 1225. Acridine Orange cell counts showed that cells were present throughout the sediment column, ranging between approx. 3 × 10^5^ and 3 × 10^6^ cells/cm^3^. These numbers were at the low end of the cell counts for the organic-rich Peru Margin and Peru Trench ODP Sites (approx. 5 × 10^6^ to 10^8^ cells/cm^3^, with some higher outliers [18]). Attenuated respiration rates in this organic-poor sediment were reflected in deep penetration of electron acceptors; for example, porewater nitrate concentrations decreased from ca. 33 µm to the detection limit within the upper 1.5 m of the top sediment core [18]. Characteristics for Leg 201 sites are tabulated in Table S1.

**Sample collection**. Deep-sea sediment was collected in February 2002 on ODP Leg 201 with JOIDES Resolution at Site 1225 in the central Pacific Ocean at 3760 m water depth. Sediment cores were retrieved by advanced piston coring (APC) and brought to the surface within 1–2 h. Freshly retrieved cores (total length 9–9.5 m) were immediately divided into 1.5 m subcores, which were immediately sliced in the ship’s cold room into whole round core samples of 5–10 cm length using an N_2_-flushed core cutting device [19]. Within 2–4 h after core retrieval, sediment samples were frozen and kept at −80 °C until processing in the home laboratory.

**DNA extraction from sediment samples.** Two approaches were used for extracting DNA from sediment samples. With direct lysis of microbial cells followed by DNA extraction from bulk sediment (Method 1), two parallel DNA extractions on a sediment sample from the uppermost 1.5 m subcore 1225C-1H1 (sample position 105–110 cm below surface, or cmbsf) yielded PCR-amplifiable DNA; extractions from other samples using this method remained unsuccessful. The sample position within the subcore represents a minimal depth estimate (1.05–1.10 mbsf), since the sediment surface is heavily perturbed during drilling, and the immediate sediment surface is lost. As an alternative DNA extraction method, prokaryotic cells were separated from the sediment sample by short sonication, followed by DNA extraction from isolated cells (Method 2). This method yielded PCR-amplifiable DNA from sample 1225C-1H6, which had previously given negative results. Subcore 1225C-1H6 represents the sediment depth range from 7.5 to 9 mbsf; thus, the sample from the 25–30 cm interval of this core segment originates from a depth of at least 7.75–7.8 mbsf.

In Method 1, genomic DNA was extracted from 10 g of deep-sea sediment sample material as follows. The sediment was suspended in 3 mL of DNA extraction buffer (200 mM NaCl, 200 mM Tris, 2 mM sodium citrate, 10 mM CaCl_2_, 50 mM EDTA, pH 8) in a 50 mL Falcon tube and freeze–thawed twice (−80 °C, 65 °C). Then, 100 µL of polyadenylic acid (polyA) (10 mg/mL), 100 µL of 6.7% sodium pyrophosphate, and 150 µL of lysozyme (100 mg/mL) were added in this order. The sediment slurry was briefly vortexed after the addition of each chemical and incubated for 40 min at 37 °C in a slowly shaking incubation chamber. Next, 50 µL of sodium dodecylsulfate (SDS) (20%) and 100 µL proteinase K (20 mg/mL) were added to the mixture followed by 3 h incubation at 55 °C with gentle shaking. The sample was extracted twice with an equal volume of phenol:chloroform: isoamylalcohol 25:24:1 (pH 8). The aqueous phase was precipitated overnight with 0.1 vol of 5 M ice-cold NaCl solution, 2.5 vol of ice-cold ethanol (96%), and 20 µg of glycogen per mL of solution (20 mg/mL stock solution). The DNA was collected by centrifugation (16,000× *g*, corresponding to 14,000 rpm in an Eppendorf 5415C tabletop centrifuge), washed in ice-cold 70% ethanol, air dried, and resuspended in 50 µL sterile distilled water. DNA extracts were purified using the Wizard Plasmid Purification Kit by Promega (Madison, WI, USA) and stored at −80 °C. A reagent blank was also processed to assess the extent of laboratory or sample cross-contamination. Outer edges of subcores were not extracted, in order to avoid contamination introduced during coring and packaging.

In Method 2, extraction was preceded by a short sonication step. Sediment samples (15 g) from subcores 1225C-1H6 and 1225C-1H1 were suspended in 4 mL of phosphate-buffered 3 M KCl (pH 7.5), 5 mL of SDS (20%) and 10 mL of sterile water, and sonicated in a water bath for 1 min. Only sample 1225C-1H6 gave a positive result in the nested PCR. Longer sonication steps (2.5 and 5 min) were also tested on both samples, with consistently negative results in all PCR experiments. The sediment slurries were spun down for 1 min at 1000 rpm, and the supernatant was extracted with Method 1.

**Amplification of 16S rRNA genes.** The primers used in this study and their references are listed in Table 1. Primer acronyms followed by f refer to forward primers; r indicates reverse primers. For amplification of archaeal 16S rRNA genes, primers ARC8f and ARC1492r (Table 1) were used initially, and the products were used as template in a subsequent nested PCR. In the nested PCR, primers ARC21f and ARC915r were used for clone library construction, while primers ARC21f-GC (primer ARC21F with GC-clamp 5′-CGC CCG CCG CGC GCG GCG GGC GGG GCG GGG GCA CGG GGG G-3′ [24] attached at the 5′-end) and ARC519r were used for denaturant gradient gel electrophoresis (DGGE) analysis.

PCR mixtures contained 1 µL of 1:10 diluted DNA extract, 2 µL of each primer (10 pmol/µL), 2 µL of deoxynucleoside triphosphates (dNTPs) (10 mM each), 5 µL of 10× PCR buffer (final concentration 1.5 mM MgCl_2_), 2 µL of bovine serum albumin (BSA, 10 mg/mL), 0.15 µL of Taq polymerase (5 U/µL) (Promega, Madison, WI, USA) and sterile water to a final volume of 50 µL. The PCR amplification was performed using an iCycler (BioRad, Hercules, CA, USA). The PCR mix was incubated at 94 °C for 4 min, followed by 35 cycles of denaturation at 94 °C for 1 min, annealing at different temperatures (Table 1) for 1 min, and extension at 72 °C for 1 min. The PCR amplification ended with a single 10 min extension step at 72 °C. The PCR products were evaluated by gel electrophoresis on 1.5% agarose gels stained with ethidium bromide (0.5 mg/L). Successful PCR amplifications for archaea were obtained with 1:10 diluted DNA extract; higher dilutions und undiluted DNA extracts did not consistently produce PCR amplicons.

**DGGE analysis.** DGGE was performed using a DCode system (Bio-Rad, Hercules, CA, USA) as previously described [24]. PCR samples were loaded onto 6% (wt./vol) polyacrylamide gels in 0.5× TAE (20 mM Tris, 10 mM acetate, 0.5 mM EDTA [pH 8]), cast with a gradient of 20%–70% denaturing agent (100% denaturant contains 7 M urea and 40% formamide). Electrophoresis was performed at 60 °C for 2 h at 250 V on a Biorad DCode system. After electrophoresis, the DGGE gels were stained for 30 min in an ethidium bromide bath (0.5 mg/L in 0.5× TAE buffer) and destained for at least 10 min in 0.5× TAE buffer. The gels were photographed on a UV transillumination table (Spectroline^®^, BI-O-VISION, New Haven, CT, USA) with a digital camera (Kodak EDAS 290). Archaeal DGGE analysis had to be limited to sample 1225-1H1, due to persistent, false-positive DNA amplicons in the negative controls of 1225-1H6. Archaeal DGGE bands of sample 1225-1H1 were selected for sequencing, punched from the gel with sterile scalpels and placed in sterile vials containing 100 µL of sterilized deionized water. After overnight incubation at 4 °C, 2 µL of the eluate was used as template in a PCR with the DGGE primers. 

**Cloning of PCR products.** Clone libraries were prepared by using the TOPO^®^ XL PCR cloning kit (Invitrogen, Carlsbad, CA, USA). PCR products were ligated to pCR^®^-XL-TOPO^®^ vectors, which were transformed into *E. coli* JM 109 cells by electrophoresis. After streaking on Luria broth (LB)-agar plates and incubation at 37 °C according to the manufacturer’s instructions, individual clones were transferred to liquid LB-medium and grown overnight on a shaking table at 37 °C. Plasmid extraction and purification was performed with the Wizard Plasmid Purification kit (Promega). To test cloning success, 6–10 randomly picked clones were screened for inserts by PCR amplification with primers M13F (5’-GTAAAAACGACGGCCAG-3’) and M13R (5’-CAGGAAACAGCTATGAC-3’) [25]. The PCR contained 1 µL of template, 2.5 µL of each primer (10 pm/µL), 2.5 µL of dNTPs (10 mM), 2.5 µL of 10× PCR buffer (final concentration 1.5 mM MgCl_2_), 0.15 µL of Taq polymerase (5 U/µL), and sterile water to a final volume of 25 µL. The PCR reaction included an initial denaturation step at 94 °C for 2 min followed by 25 cycles of denaturation at 94 °C for 30 s, annealing at 55 °C for 30 s, and extension at 72 °C for 30 s. The step at 72 °C was extended to 5 min.

**Sequencing.** Sanger sequencing using fluorescent dideoxy terminators was performed at the Glaxo Sequencing Center of the University of North Carolina at Chapel Hill. Reamplified DGGE bands were purified prior to cloning by using the MOBIO PCR cleanup kit (MOBIO, Solana Beach, CA, USA) according to the manufacturer’s protocol. About 50 ng of template and 10 pmol of primers ARC21f and ARC519r were used in the sequencing reaction. Clone libraries were sequenced using 10 pmol of primers M13F and M13R in reactions with 0.7 µg of plasmid template.

**Phylogenetic analysis.** Sequence data were analyzed by BLAST against the entire GenBank database [26]. Sequences were aligned in a first step with the program Multalign (http://prodes.toulouse.inra.fr/multalin/), followed by manual alignment with SeqPup v0.6 [27]. Primer sequences were excluded from alignments and phylogenies. The sequences were checked for chimeras using the online detection tool Bellerophon [28], complemented by manual inspection of the alignment and tree building using partial sequences. Phylogenetic analyses were performed with PAUP4.0* [29] using maximum likelihood distances under minimum evolution optimality criteria; empirically estimated base frequencies and transition/transversion frequencies were used. Bootstrap analyses are based on 200 replicates.

**Nucleotide sequence accession numbers.** The archaeal 16S rDNA sequences obtained in this study are available in GenBank under accession numbers AY800198 to AY800233 and DQ186521 (clone 1CD3). The 16S rDNA sequences of the archaeal DGGE fragments have GenBank accession numbers DQ137876 to DQ137881.

## 3. Results

**Extraction of PCR-amplifiable DNA.** DNA extraction was attempted from numerous samples throughout the sediment column, using Methods 1 and 2 as described above. When the DNA extracts were evaluated by electrophoresis on 1% agarose gels, DNA concentrations were too low to be visualized by ethidium bromide staining. Nevertheless, archaeal DNA was successfully amplified from the two shallowest samples (1225C-1H1 and 1225C-1H6). Method 1 worked in two parallel extractions and PCR amplifications of archaeal DNA from sample 1225C-1H1, which resulted in 25 and 29 archaeal sequences, respectively. The clones obtained from parallel DNA extractions and amplification from sample 1225C-1H1 differed in relative abundance of phylogenetic groups. The majority of the clones from the first DNA extraction (clone names beginning with 1C) are affiliated with different subgroups of Marine Group 1 (MG-I), whereas most clones from the second DNA extraction (beginning with 1A) were members of Deep-Sea Archaeal Group/Marine Benthic Group B (DSAG/MBG-B) (Table 2).

Since the same DNA extraction method was used, these variable results could reflect either stochastic PCR bias due to random amplification from a small number of DNA templates [29] or spatial heterogeneity in the sediment sample. PCR-amplifiable DNA was extracted using Method 2 from sample 1225C-1H6, and a total of 26 clones were sequenced. DNA extracted with Method 1 from sample 1225C-1H6 gave negative results in the nested PCR.

**Figure 2:**
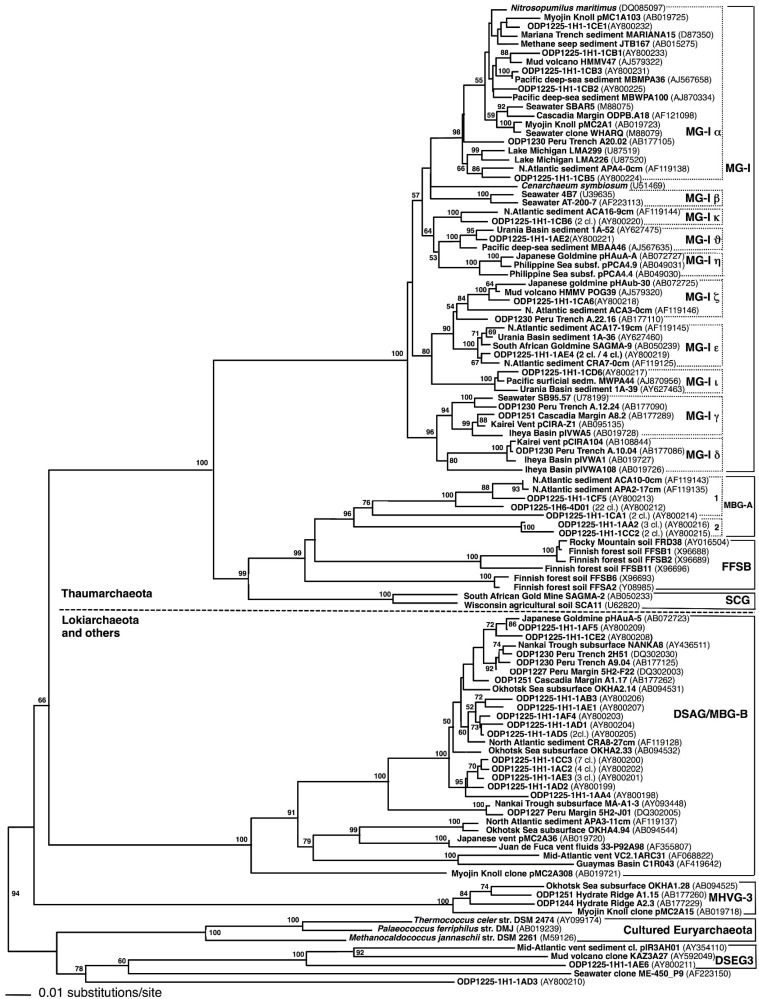
Phylogenetic tree (minimum evolution) of archaeal 16S rRNA genes (*E. coli* positions 25–914) from clone libraries of sediment samples 1225C-1H1 and 1H6. Sequences in the phylogeny that represent multiple clones are annotated with clone numbers in parentheses, between clone name and GenBank number. The annotation “2 cl/4 cl” indicates that clone ODP 1225-1H1-1AE4 stands for 2 clones obtained from sample 1225-1H1 and 4 clones from sample 1225-1H6. Bootstrap numbers are based on 200 resamplings. Euryarchaeota (cultured euryarchaeota and the euryarchaeotal lineage DSEG3) were placed as the outgroup.

**Marine Group I.** Most of the detected archaeal phylotypes were affiliated with Marine Group I (MG-I) within the Thaumarchaeota [30]. The MG-I clones from Site 1225 could not be accommodated solely by the pelagic MG-I subgroups α, β, and γ, defined originally by comparative sequence analyses of MG-I *Archaea* from different oceans and water depths [31]. Subgroup MG-Iα included clones from all water depths, but especially from the upper 200 m of the water column [31]; it also accounted for almost half of the MG-I clones obtained from Site 1225 (Figure 2). Subgroup MG-Iγ harbored mostly deep-water phylotypes collected between 450 and 3000 m water depth [31]. Following the Greek alphabet, the additional clusters δ (delta), ε (epsilon), η (eta), and ζ (zeta) were subsequently proposed to accommodate the increasing diversity of the MG-I *Archaea* from hydrothermal vents and subsurface sediments [3,32]. Three additional clusters of sediment MG-I phylotypes (κ, kappa; ι, iota; ν, upsilon) were defined to accommodate novel MG-I phylotypes from Site 1225 and other marine sediments and habitats, including coastal and open ocean waters worldwide [22,31], Lake Michigan sediment [33], deep-sea hydrothermal vents [32,34], subsurface water of a Japanese epithermal goldmine [35], deep-sea subseafloor sediments [36], and from within the walls of an active deep-sea sulfide chimney on the Juan de Fuca Ridge [37]. The same MG-I subgroups were used subsequently for fine-scale phylogenetic placement of MG-I archaea from organic-lean sediments in the South Pacific [4]. Interestingly, the MG-I phylotypes from Sites 1225, 1231 and the South Pacific showed very little phylogenetic overlap with the MG-I clones from deep subsurface sediments of organic-rich continental margin sites (Peru Trench Site 1230 and Cascadia Margin Site 1251), which fell mostly into MG-I group γ [11].

**Deep-Sea Archaeal group (DSAG).** Altogether, 24 clones from sample 1225C-1H1 belonged to the Marine Benthic Group B (MBG-B) [38], synonymous with Deep-Sea Archaeal Group [34,35], and recently renamed as the distinct phylum Lokiarchaeota [39]. This uncultured archaeal lineage has been found in a wide range of marine sediments and hydrothermal vent samples [1,6,40], but appears to prefer organic-rich, reducing sediments [1]. While most deeply branching members of this lineage were generally found at hydrothermal vents [20,34,41], a tightly clustering branch of DSAG/MBG-B *Archaea* includes predominantly sequences from cold marine sediments, surficial as well as subsurface [3,35,36,38,42] (Figure 2). All DSAG/MBG-B phylotypes from Site 1225 fell into this cluster of marine sediment phylotypes, with 100% bootstrap support (Figure 2). The second sediment sample, 1225C-1H6, did not yield DSAG/MBG-B sequences.

**Marine Benthic Group A (MBG-A).** A single sequence from sample 1225C-1H1 (AY800213), and 22 nearly identical clones from sample 1225C-1H6, represented by clone AY800212, grouped with Marine Benthic Group A (MBG-A) (Figure 2). This uncultured archaeal cluster was initially described from cold continental slope and deep-sea sediments in the North Atlantic [38]; its members constitute subgroup MBG-A1 [5]. Seven clones from sample 1225C-1H1 (represented by AY800214, AY800215, and AY800216) formed two sister lineages to MBG-A (Figure 2). The latter two clones belonged to subcluster MBG-A2, also consistently represented by phylotypes from oligotrophic marine sediments [5]. Other relatives of MBG-A are members of the Finnish Forest Soil Boreal Group (FFSB) [43] found in boreal forests. These lineages and MBG-A share a common root with good bootstrap support that separates them from their closest sister group, the Soil Crenarchaotal Group (SCG) from agricultural soils and from geothermal water in South African gold mines [44]. In turn, MBG-A, FFSB, and SCG together share bootstrap support of 99%, and constitute a sister lineage of MG-I within the phylum Thaumarchaeota (Figure 2).

***Euryarchaeota.*** Two sequences were members of the *Euryarchaeota* (Figure 2). Together with marine sequences from Mediterranean mud volcano sediments (AY592049) and from metal-rich sediments in the vicinity of hydrothermal vents (AY354110), clone 1AE6 formed a monophyletic group, termed Deep-Sea Euryarchaeotal Group 3 [5]. Sequence 1AD3 could not be assigned to a specific lineage.

**DGGE analysis.** The DGGE profile of sample 1225C-1H1 yielded eight bands; after excising and eluting the DGGE bands, six of these could be successfully reamplified and sequenced. The sequences of DGGE bands 1 and 2 were related to MBG-A clones, respectively, whereas the sequences of bands 4, 5, 7, and 8 fell within the MG-I archaea; bands 3 and 6 could not be amplified (Figure S1). Using a different primer combination and methodology than the clone libraries, the DGGE analysis demonstrated independently that MG-I and MBG-A were among the dominant archaeal groups in the 1225C-1H1 sediment sample.

**Chimeras.** Seven archaeal 16S rRNA gene clones were recognized as likely chimeras, and were removed from the phylogeny in Figure 2. The last 85 positions of MG-I clone DQ186521 were derived from a DSAG/Lokiarchaeota phylotype. The last 60 positions of clone AY800222 deviated conspicuously from the phylogenetic consensus of the MG-Iν group. Clone AY800223 was loosely affiliated with MG-1α, but could not be placed reliably. The clone AY800226 combined a phylotype very closely related to clone AY800231 and a more distantly related phylotype within the MG-Iα group. The clone AY800227 combined a phylotype very closely related to clone AY800225 and a more distantly related phylotype within the MG-Iα group. The clones AY800229 and AY800230 within the MG-Iα group consisted of up to ca. 60% of a distinct phylotype related to clone AY800220 in the MG-Iκ group. Clone AY800228, originally positioned within MG-1α, was identified as a chimera with MG-Iε and ζ phylotypes. Excluding these sequences from the phylogeny improved the bootstrap support of especially MG-Iα considerably, from near 50% to 98% (Figure 2). We call attention to these chimeras and document them clone by clone, since they occur almost entirely at the level of mutually closely related MG-I subclusters that are separated only by max. 3%–4% sequence divergence, close to the threshold for defining operational taxonomic units [4]. A fine-grained, and at the same time robust, phylogenetic framework that resolves the actually existing microclusters within a phylogenetic lineage facilitates their detection.

## 4. Discussion

**Downcore trends in archaeal detection.** Although deep sediment samples were repeatedly extracted using different approaches, only two samples from the upper sediment column, 1225C-1H1 and 1H6, yielded PCR-amplifiable DNA. Similar difficulties of obtaining PCR-amplifiable archaeal DNA from deeper sediment layers occurred in the South Pacific Gyre, most extremely at Site SPG11 where only the upper 30 cm of the sediment column yielded workable material [4]. Quantifications of archaeal populations in organic-rich sediment cores from the Peru Margin and Peru Trench (ODP Sites 1227 and 1230) using 16S rDNA-targeted q-PCR showed that the highest densities of archaeal 16S rRNA gene copies were found near the sediment surface, and decreased by several orders of magnitude towards deeper sediment horizons [10]. Similar patterns of rapidly decreasing archaeal gene numbers were found by qPCR analysis of the oligotrophic sediment column at North Pond, a sedimented basin near the mid-Atlantic Ridge [45]. These trends are broadly consistent with the downcore profiles of direct cell counts in oligotrophic sediments in the South Pacific Gyre, with the caveat that the method does not distinguish between archaea and bacteria [2,46].

**Figure 3:**
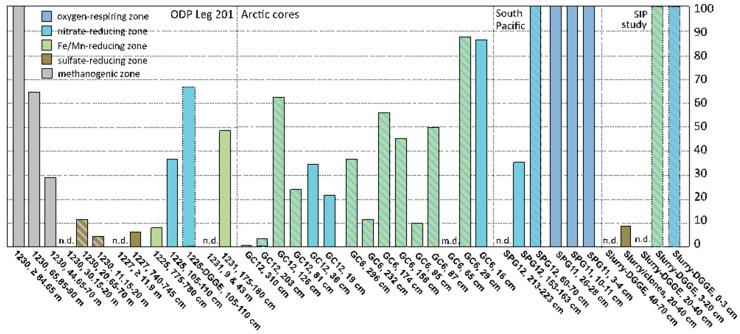
Bar diagram showing percentages of Marine Group I (MG-I) representation in archaeal 16S rRNA gene clone libraries, denaturant gradient gel electrophoresis (DGGE) and pyrosequencing profiles for marine sediment core samples, color-coded by electron acceptor availability. Color stripes indicate evidence for coexisting electron acceptors or pathways, or ambiguity in the data. Ocean Drilling Program (ODP) site numbers represent Equatorial Pacific Site 1225 [this study], Peru Margin Site 1227 [11], Peru Basin Site 1231 [3], and Peru Trench Site 1230 [11]. Arctic Ocean cores GC12 and GC6 from sediment cores near the hydrothermally active Gakkel Ridge were analyzed by 16S rRNA gene pyrosequencing [6]. SPG11 and SPG12 refer to South Pacific Gyre core SPG11 and South Pacific core SPG12 [4,5]. DGGE slurry data refer to DGGE results from stable carbon isotope probing experiments with sediments from the Severn Estuary, UK [48]. n.d., not detected; m.d., missing data. Data underlying this bar diagram are compiled in Table S1.

**Range and diversity of MG-**I **archaea in marine sediments.** Detecting members of the MG-I *Archaea* at Site 1225 and in many other benthic marine sediments [1,4,6] raises the question whether these *Archaea* can survive and grow in marine sediments, potentially by nitrate reduction or other anaerobic metabolisms, or whether they represent sedimentation from pelagic, aerobic populations in the water column. Since MG-I *Archaea* are highly abundant in ocean water, and constitute the dominant component of bacterial and archaeal plankton in the mesopelagic water column [47], they could contribute to the archaeal populations in near-surface sediments. However, if their range extends into anoxic subsurface sediments, MG-I archaea are unlikely to represent depositional remnants from the oxygenated water column, and constitute indigenous populations of marine sediments; this interpretation would also be consistent with the detection of specific sediment MG-I subclusters. A compilation of case studies (incl. the results of this study) shows that the distribution of MG-I archaea in marine sediment cores appears to be linked to the availability of the electron acceptors oxygen and nitrate in porewater; MG-I archaea can also extend below the zone of porewater nitrate depletion if nitrate remains available adsorbed to the solid phase (Figure 3).

In South Pacific sediments of core SPG12, MG-I archaea persisted at 1.53–1.63 mbsf but were no longer detectable at 2.13–2.23 mbsf; porewater nitrate was entirely consumed near 2.5 mbsf [5]. In sediments of the Peru Basin at ODP Site 1231, MG-I sequences were obtained only from a sample at 1.8 m depth in the near-surface core [3]; porewater nitrate at above-background concentrations was detected within the upper 1 m [49]. At Site 1225, multiple MG-I subgroups were found in the upper sediment sample at 1.05–1.10 mbsf where porewater nitrate remained available. Only MG-I subgroup ε was detected in the deeper sample at 7.75–7.80 mbsf, below the penetration depth of porewater nitrate. In a similar case from subsurface sediments of the Arctic Ocean, MG-I archaea occurred in two gravity cores with a length of ca. 3 m each (GC6 and GC12 in Figure 3). These cores were depleted in porewater nitrate below 30–40 cmbsf, but rich in Mn^2+^ and Fe^2+^; however, nitrate remained available in the solid phase where it persisted, presumably due to absorption to mineral particles [6]. Interestingly, MG-I archaea were found in DGGE analyses of stable carbon isotope probing (SIP) incubations with ^13^C-CO_2_, inoculated with oxic and anoxic surficial intertidal sediments from the Severn Estuary, UK [48]. When sulfate-reducing sediments below 20 cm depth were used, MG-I archaea were no longer detected by DGGE but they appeared as a minority population in a clone library analysis [48]. In a well-documented but so far isolated case, MG-I archaea were detected consistently in clone library analyses of sulfate-reducing and methanogenic, even hydrate-containing deep subsurface sediments of ODP Site 1230 in the Peru Trench [11], with the caveat that MG-I archaea could not be detected in an independent archaeal community analysis of the same site using reverse transcription of RNA [12].

Compiling the overall contribution of MG-I archaea to clone libraries, DGGE profiles, and pyrosequencing surveys of marine sediments in relation to oxic, nitrate-reducing, metal-reducing, sulfate-reducing, or methanogenic geochemical regime reveals the persistence of MG-I archaea under oxic and nitrate-reducing conditions, the latter overlapping with metal-reducing conditions; they are rarely seen under sulfate-reducing conditions, and their abundant detection in methanogenic sediments of ODP Site 1230 stands out as unique (Figure 3, and Table S1).

**ODP Site 1225 and its archaeal community in comparative perspective.** Extending a previous comparative study [1], marine subsurface sediments can be placed into different categories of organic carbon content and redox status in the subsurface sediment column (data tabulated in Table S1). Extremely organic-depleted sediments, for example those of the South Pacific Gyre [50], are characterized by a total organic carbon [TOC] content below 0.05 wt. % and contain porewater oxygen and nitrate throughout their sediment column down to basement basalt; clone libraries from this sediment type are available from SPG 11 [4]. The next category, organic carbon-depleted sediments with a total organic carbon content below 0.5 wt. %, are characterized by oxygen and nitrate depletion within a few meters, followed by metal reduction; yet porewater sulfate extends largely unchanged to basement and sulfide does not accumulate. With TOC content between 0.2 and 0.4 wt. %, ODP Site 1225 represents this category [18]. SPG12 on the edge of the South Pacific Gyre [5] and ODP site 1231 [3] also provide good examples for organic carbon-depleted sediments. Coring sites in the South China Sea appear to fall into this category as well, with the caveat that nitrate concentrations are not available (Table S1). The well-documented Arctic Basin cores GC6 and GC12 [6] are at the organic-rich end of this category; some TOC values exceed >1%, due to the shallow depth (ca. 3 m) of these gravity cores (Table S1); however, the TOC content of subsurface sediments below 3 mbsf remains unknown. For the following category, intermediate organic-depleted sediments, an upper TOC concentration threshold of 2 wt. % was selected as cutoff point, based on the examples Table S1. These sediments are characterized by surficial oxygen and nitrate penetration, and significant sulfate depletion and sulfide accumulation in the deep sediment column. ODP Site 1226 in the Equatorial Pacific near Galapagos and IODP Site 308 in Brazos Basin, Gulf of Mexico, provide examples (Table S1). At last, organic carbon-rich sediments with TOC concentrations above 2% are characterized by oxygen and nitrate depletion within a few centimeters, and sulfate depletion leading to methanogenesis in the deeper sediment column. The Peru Margin sediments at ODP Sites 1227, 1228, and 1229, as well as Peru Trench Site 1230 provide examples [1,11,12].

While this simple scheme is open to improvement as the database matures, it corresponds to systematically changing archaeal communities (Table S1): Under conditions of extremely low sedimentation and organic carbon input, most archaeal taxa become undetectable and their apparent depth extent decreases, until sequencing surveys recover only MG-I archaea from the fully oxidized sediment column or extremely organic-depleted sediments [4]. In the organic-depleted category, other archaeal groups such as DSAG/MBG-B, Marine Benthic Group A, and Deep-Sea Euryarchaeotal groups are added or superimposed on the MG-I archaea, in particular below the oxic and nitrate-reducing zone ([3,5] this publication); this trend continues in the moderately organic-depleted category (Table S1). Sediments of the organic carbon-rich category harbor archaeal communities that are dominated by anaerobic lineages that prefer or require reducing habitats, such as Bathyarchaeota, Lokiarchaeota, SAGMEG, or methanogens [11,12].

As an interesting feature of organic-depleted but non-extreme open ocean locations, extended porewater gradients of electron acceptors and small amounts of sedimentary organic carbon allow the coexistence of physiologically and phylogenetically distinct archaeal lineages, as observed here at ODP Site 1225. These widely occurring non-extreme seafloor sediments provide a natural laboratory for fine-scale analyses correlating the downcore changes of microbial communities and their geochemical environments, on a scale of several meters that is compact enough for sampling (for example, by gravity coring) but sufficiently extended to provide spatially resolved sediment samples. Such studies could further explore correlations of individual MG-I sediment clusters with specific biogeochemical niches in oxic, nitrate-reducing, or metal-reducing sediments.

## Figures and Tables

**Table 1:** Archaeal 16S rDNA primers.

Primer	Sequence	Annealing Temperature (°C)	Reference
ARC8f	5′-TCCGGTTGATCCTGCC-3′	55	[20]
ARC1492r	5′-GGCTACCTTGTTACGACTT-3′	55	[21]
ARC21f	5′-TTCCGGTTGATCCYGCCGGA-3′	65	[22]
ARC915r	5′-GTGCTCCCCCGCCAATTCCT-3′	65	[23]
ARC344f	5′-AYGGGGYGCASCAGGSG-3′	65	[24]
ARC519r	5′-GGTDTTACCGCGGCKGCTG-3′	65	[24]

**Table 2:** Numbers of 16S rRNA gene clones affiliated with major archaeal groups retrieved after DNA extractions from Site 1225 sediment samples. The two replicate extractions for sediment sample 1225-1H1 are separated into two data columns.

Affiliation	1225C-1H1 Extraction 1 Method 1	1225C-1H1 Extraction 2 Method 1	1225C-1H6 Method 2
Marine Group I	17	3	4
Marine Benthic Group A	5	3	22
DSAG/Marine Benthic Group B	3	21	-
Euryarchaeota	-	2	-
Sum	25	29	26

## Supplementary Materials

The following are available online at www.mdpi.com/2076-2607/4/3/32/s1, Figure S1: Annotated DGGE analysis of sediment sample 1225C-1H1 with bands 1 to 8, with phylogenetic identification, Table S1: Site characteristics for marine subsurface sediments, and MG-1 archaea.
