# Supplementary material for: Phylogenetic Characterization of Marine Benthic Archaea in Organic-Poor Sediments of the Eastern Equatorial Pacific Ocean (ODP Site 1225)
Source: Microorganisms. 2016 Sep 6;4(3):32. doi: 10.3390/microorganisms4030032 (PMC5039592; doi:10.3390/microorganisms4030032)
Supplement: Supplementary file 1 [file microorganisms-04-00032-s001.zip › microorganisms-04-00032-supplementary Figure S1.pdf]

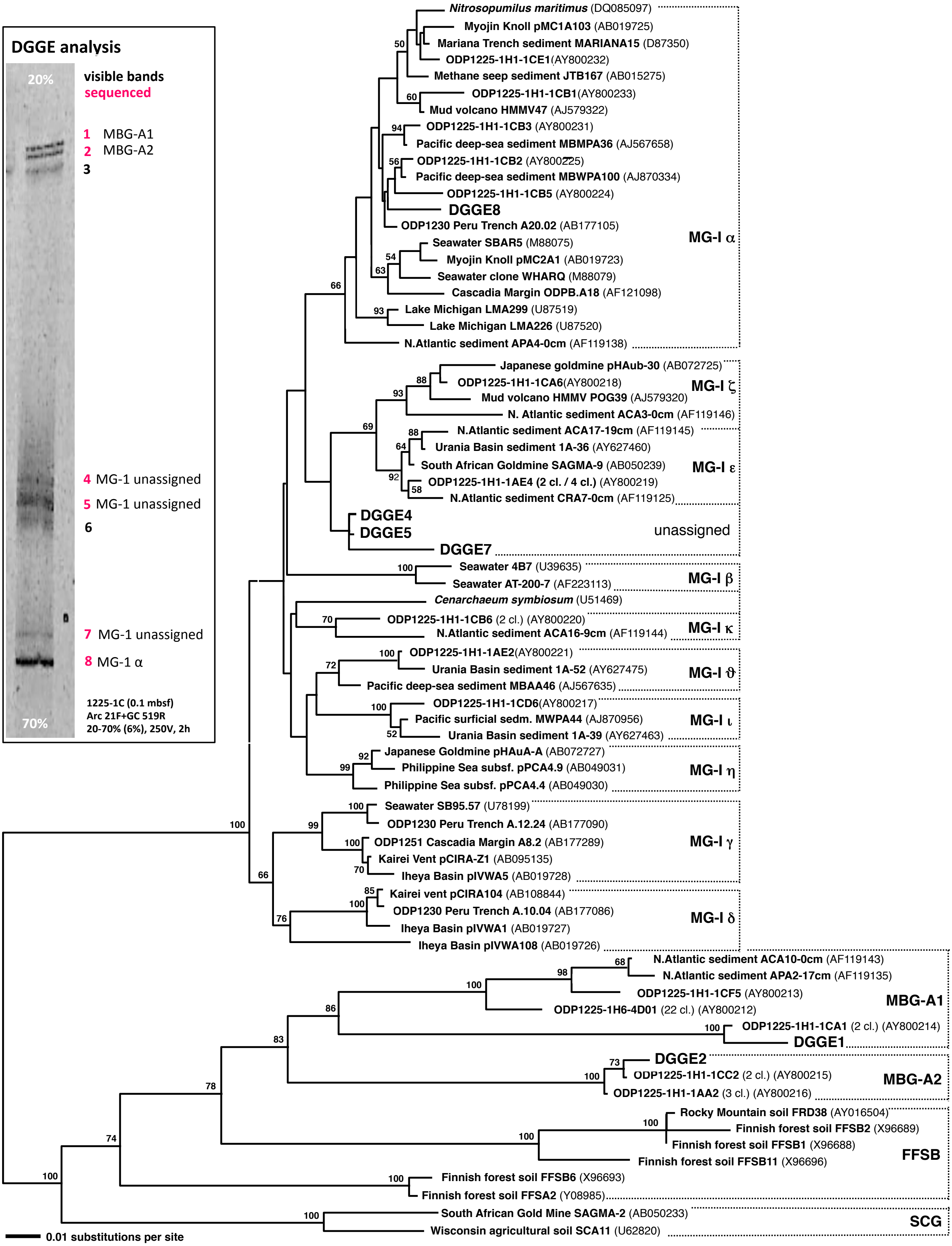

DGGE analysis
20%
visible bands
sequenced
1 MBG-A1
2 MBG-A2
3
4 MG-1 unassigned
5 MG-1 unassigned
6
7 MG-1 unassigned
8 MG-1 α
70%
1225-1C (0.1 mbsf)
Arc 21F+GC 519R
20-70% (6%), 250V, 2h
Nitrosopumilus maritimus (DQ085097)
Myojin Knoll pMC1A103 (AB019725)
Mariana Trench sediment MARIANA15 (D87350)
ODP1225-1H1-1CE1(AY800232)
Methane seep sediment JTB167 (AB015275)
ODP1225-1H1-1CB1(AY800233)
Mud volcano HMMV47 (AJ579322)
ODP1225-1H1-1CB3 (AY800231)
Pacific deep-sea sediment MBMPA36 (AJ567658)
ODP1225-1H1-1CB2 (AY800225)
Pacific deep-sea sediment MBWPA100 (AJ870334)
ODP1225-1H1-1CB5 (AY800224)
DGGE8
ODP1230 Peru Trench A20.02 (AB177105)
Seawater SBAR5 (M88075)
Myojin Knoll pMC2A1 (AB019723)
Seawater clone WHARQ (M88079)
Cascadia Margin ODPB.A18 (AF121098)
Lake Michigan LMA299 (U87519)
Lake Michigan LMA226 (U87520)
N.Atlantic sediment APA4-0cm (AF119138)
MG-I α
Japanese goldmine pHAub-30 (AB072725)
ODP1225-1H1-1CA6(AY800218)
Mud volcano HMMV POG39 (AJ579320)
N. Atlantic sediment ACA3-0cm (AF119146)
MG-I ζ
N.Atlantic sediment ACA17-19cm (AF119145)
Urania Basin sediment 1A-36 (AY627460)
South African Goldmine SAGMA-9 (AB050239)
ODP1225-1H1-1AE4 (2 cl. / 4 cl.) (AY800219)
N.Atlantic sediment CRA7-0cm (AF119125)
MG-I ε
DGGE4
DGGE5
DGGE7
unassigned
Seawater 4B7 (U39635)
Seawater AT-200-7 (AF223113)
MG-I β
Cenarchaeum symbiosum (U51469)
ODP1225-1H1-1CB6 (2 cl.) (AY800220)
N.Atlantic sediment ACA16-9cm (AF119144)
MG-I κ
ODP1225-1H1-1AE2(AY800221)
Urania Basin sediment 1A-52 (AY627475)
Pacific deep-sea sediment MBAA46 (AJ567635)
MG-I ϑ
ODP1225-1H1-1CD6(AY800217)
Pacific surficial sedm. MWPA44 (AJ870956)
Urania Basin sediment 1A-39 (AY627463)
MG-I ι
Japanese Goldmine pHAuA-A (AB072727)
Philippine Sea subsf. pPCA4.9 (AB049031)
Philippine Sea subsf. pPCA4.4 (AB049030)
MG-I η
Seawater SB95.57 (U78199)
ODP1230 Peru Trench A.12.24 (AB177090)
ODP1251 Cascadia Margin A8.2 (AB177289)
Kairei Vent pCIRA-Z1 (AB095135)
Iheya Basin pIVWA5 (AB019728)
MG-I γ
Kairei vent pCIRA104 (AB108844)
ODP1230 Peru Trench A.10.04 (AB177086)
Iheya Basin pIVWA1 (AB019727)
Iheya Basin pIVWA108 (AB019726)
MG-I δ
N.Atlantic sediment ACA10-0cm (AF119143)
N.Atlantic sediment APA2-17cm (AF119135)
ODP1225-1H1-1CF5 (AY800213)
ODP1225-1H6-4D01 (22 cl.) (AY800212)
ODP1225-1H1-1CA1 (2 cl.) (AY800214)
DGGE1
MBG-A1
DGGE2
ODP1225-1H1-1CC2 (2 cl.) (AY800215)
ODP1225-1H1-1AA2 (3 cl.) (AY800216)
MBG-A2
Rocky Mountain soil FRD38 (AY016504)
Finnish forest soil FFSB2 (X96689)
Finnish forest soil FFSB1 (X96688)
Finnish forest soil FFSB11 (X96696)
FFSB
Finnish forest soil FFSB6 (X96693)
Finnish forest soil FFSA2 (Y08985)
South African Gold Mine SAGMA-2 (AB050233)
Wisconsin agricultural soil SCA11 (U62820)
SCG
0.01 substitutions per site
